# Pancreatic cancer: Circulating Tumor Cells and Primary Tumors show Heterogeneous *KRAS* Mutations

**DOI:** 10.1038/s41598-017-04601-z

**Published:** 2017-07-03

**Authors:** Birte Kulemann, Stephanie Rösch, Sindy Seifert, Sylvia Timme, Peter Bronsert, Gabriel Seifert, Verena Martini, Jasmina Kuvendjiska, Torben Glatz, Saskia Hussung, Ralph Fritsch, Heiko Becker, Martha B. Pitman, Jens Hoeppner

**Affiliations:** 1 Center for Surgery, Department of General and Visceral Surgery, Medical Center University of Freiburg, Freiburg, Germany; 2 Institute for Surgical Pathology, Medical Center University of Freiburg, Freiburg, Germany; 3 Tumorbank Comprehensive Cancer Center, Medical Center University of Freiburg, Freiburg, Germany; 4 Department of Pathology & Andrew L. Warshaw, MD Institute for Pancreatic Cancer Research, Massachusetts General Hospital/Harvard Medical School, Boston, MA USA; 5 Center of Medicine, Department of Medicine I, Medical Center University of Freiburg, Freiburg, Germany; 6 Faculty of Medicine, University of Freiburg, Freiburg, Germany; 7 German Cancer Consortium (DKTK) and German Cancer Research Center (DKFZ), Heidelberg, Germany

## Abstract

Pancreatic ductal adenocarcinoma (PDAC) is a devastating disease. Circulating tumor cells (CTC) in the blood are hypothesized as the means of systemic tumor spread. Blood obtained from healthy donors and patients with PDAC was therefore subject to size-based CTC-isolation. We additionally compared Kirsten rat sarcoma viral oncogene homolog (*KRAS*) mutations in pancreatic CTC and corresponding tumors, and evaluated their significance as prognostic markers. Samples from 68 individuals (58 PDAC patients, 10 healthy donors) were analyzed; CTCs were present in patients with UICC stage IA-IV tumors and none of the controls (p < 0.001). Patients with >3 CTC/ml had a trend for worse median overall survival (OS) than patients with 0.3–3 CTC/ml (*P* = 0.12). Surprisingly, CTCs harbored various *KRAS* mutations in codon 12 and 13. Patients with a *KRAS* ^G12V^ mutation in their CTC (n = 14) had a trend to better median OS (24.5 months) compared to patients with other (10 months), or no detectable *KRAS* mutations (8 months; *P* = 0.04). *KRAS* mutations in CTC and corresponding tumor were discordant in 11 of 26 “tumor-CTC-pairs” (42%), while 15 (58%) had a matching mutation; survival was similar in both groups (*P* = 0.36). Genetic characterization, including mutations such as *KRAS*, may prove useful for prognosis and understanding of tumor biology.

## Introduction

Pancreatic ductal adenocarcinoma (PDAC) is the fourth leading cause of cancer-related death in the United States and Europe^1,2^. Incidence almost equals mortality with a 5-year survival rate of <6%^2^. This is mostly due to its often late diagnosis at metastatic stages, its aggressive biology and only partial response to known chemotherapies^3^.

To date, most treatment decisions are made based on the tumor stage evaluated by fine needle aspiration (FNA) cytology and cross-sectional imaging, and patients are “under staged” in about 20% of the cases since metastatic disease is often only visible upon operative exploration^4^. Conventional prognostic factors such as tumor size, nodal status and perineural invasion can be evaluated only after resection and mostly confirm the poor prognosis. Even after complete tumor resection, more than 80% of the patients develop local or distant tumor recurrence^2^. These figures highlight the need for a biomarker that can improve diagnosis and staging, and that contributes to our understanding of the tumor biology.

Circulating tumor cells (CTCs) in the blood stream are thought to represent disseminated tumor cells that have detached from the primary lesion and that are undetectable by clinical imaging and inaccessible to excision. These cells have been isolated and evaluated for the diagnostic workup and treatment monitoring of various cancers, including prostate, lung, colorectal, and breast cancer^5–14^ and are thought to undergo epithelial to mesenchymal transition (EMT) to enter the blood stream and to seed in distant organs.

While CTCs have been extensively studied in the mentioned neoplasms, their significance in PDAC at various stages is not known. There is, however, emerging evidence that CTCs may also serve as a valuable tool for outcome prediction and understanding of tumor biology in PDAC^15–19^. Previous studies in PDAC used techniques that depended on the CTC capture with antibodies to epithelial cell surface antigens. They have reported low rates of CTC positivity (5–50%) and CTC positivity was associated with at least a trend to worse progression-free and overall survival^20–24^. It is of note that if EMT does play an essential role in cancer cell spread in PDAC, CTC isolation methods that rely on epithelial surface markers alone are likely to provide an incomplete capture of the cells in the blood stream and may explain at least in part the low isolation rates and discrepant results in PDAC in the past^18–21^.

Point mutations in the *Kirsten rat sarcoma viral oncogene homolog* (*KRAS*) gene are present in over 90% of PDAC cases and are thought to be an early event in the development of PDAC, already occurring in PanIN 1A lesions of the pancreas^25–27^. The mutations typically affect the hotspot codons 12 or 13. Depending on the specific amino acid substitution, the mutations differ in their associated mRNA expression patterns, biochemical activity and transforming capacity^28,29^. The underlying biological processes are yet not understood.

The objective of our prospective study was to assess the impact of CTC counts on survival and the correlations of *KRAS* mutations in CTCs and corresponding primary tumor samples in patients with PDAC. For CTC isolation, we used a simple filtration-based technique which is independent of the CTC surface. We show that patients with >3 CTC/ml tend to have a worse overall survival (OS) than patients with 0.3–3 CTC/ml and that the *KRAS* mutations identified in CTCs or primary tumor may differ within the same patient.

## Material and Methods

### Patient selection

Patients with histologically-proven PDAC who were treated with either tumor resection or palliative bypass at our institution were included. Additionally, healthy donor blood was spiked by adding varying known quantities of PDAC cells to re-test the sensitivity of detection as partially previously described^16^. All experiments were performed in accordance with guidelines and regulations of good research practice. We confirm that all experimental protocols were approved by the institutional committee (independent Ethics Committee University of Freiburg). Written informed consent was obtained from each patient.

### CTC isolation method

**Figure 1:**
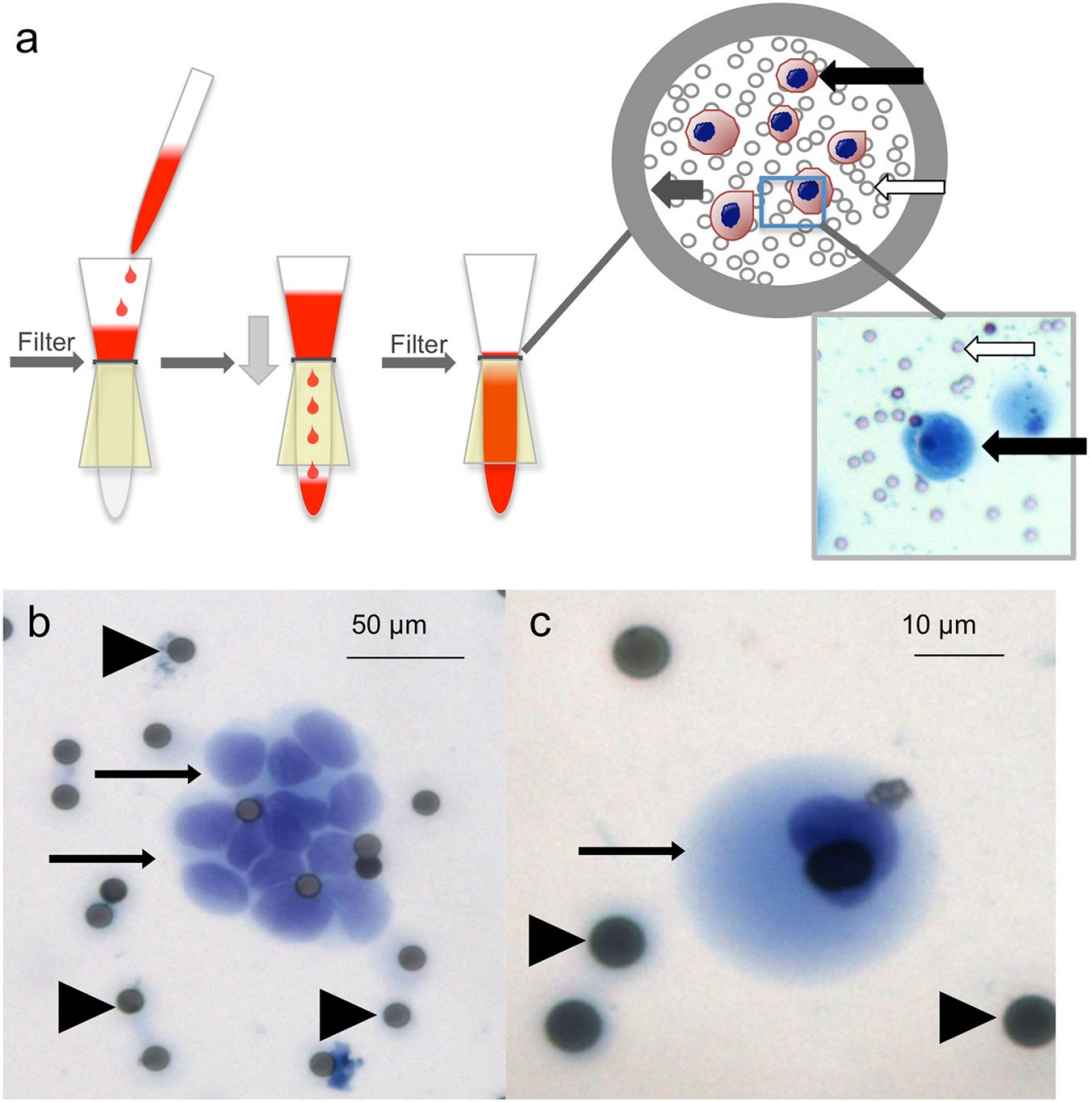
(**a**) Isolation method of CTC. White arrows point at filter pores, black arrows at “CTC” grey arrow points at the metal rim of the filter. Examples of CTC in patients with PDAC. (**b**) CTC cluster (patient 24), the scale bar represents 50 µm C single CTC (patient 23) the scale bar represents 10 µm. Arrows point at CTC, arrowheads at filter pores.

Three ml of peripheral blood samples were processed through ScreenCell® filtration devices (Paris, France) according to the manufacturer´s instructions, as previously described^16,17^, to capture CTCs within three hours of draw. The ScreenCell^®^ system is an isolation-by-size method; the devices are fitted with microfilters that capture the cells on small metal-rimmed filters via low-pressure vacuum-filtration (Fig. 1a). Cell cytology was visualized with Giemsa staining, and DNA was isolated from cells captured from 6﻿ ml blood on a parallel filter, the ScreenCell^®^ MB device for DNA isolation. Control blood samples were obtained from healthy volunteers.

### Cytological characterization of CTC

An experienced pancreatic cytopathologist (MBP) and two pathologists (PB, ST) evaluated the stained filters blinded to the histological diagnosis. The cells were categorized as positive, “suspicious”, or “negative” for malignant CTC based on established cytomorphological features for PDAC^30^. The results were evaluated independently. If the results showed inter-observer difference, the third observer re-evaluated the specimen and observers came to a final agreement.

Cells considered positive or diagnostic of malignant CTCs were epithelioid cells with markedly enlarged (8–20x filter pore size) irregular, hyperchromatic nuclei and scant, well-defined cytoplasm or smaller epithelioid cells (2–7x filter pore size) with round to oval nuclei, occasional nuclear groove and ill-defined but visible cytoplasm in small or large clusters (cluster and single cell Fig. 1). Cells suspicious for malignant CTCs were enlarged, clumped cells with molded nuclei but poorly-defined or absent cytoplasm. CTC were counted by three independent reviewers and the number of CTCs was divided by 3 to estimate the number of CTC/ml.

### Genotyping of *KRAS* in CTC samples and primary tumor

For genetic analysis of CTC, six ml of blood were filtered using the ScreenCell^®^ MB device according to the manufacturer’s instructions. DNA of cells on the filter was isolated using the Qiagen Purgene core kit (QIAGEN, Hilden, Germany). Tumor samples were randomly obtained from an unspecific part of the tumor and micro-dissected by a technician. Due to tissue availability, only one region of the tumor was analyzed. Tumor DNA was extracted from paraffin-embedded samples via the QIAamp DNA FFPE Tissue Kit (QIAGEN, Hilden, Germany). The mutational status of *KRAS* codons 12 or 13 was determined using a peptide nucleic acid (PNA) and polymerase chain reaction (PCR)-based assay, as previously described^16,31^.

Briefly, this PNA clamp anneals to the wild-type area of the *KRAS* gene and prevents amplification of the *KRAS* wild-type DNA sequences. Thus, mutations in the *KRAS* gene can be identified even in very low concentrations of 2 cancer cells/ml blood^16^. PCR products were electrophoresed on a 4% agarose gel. In the presence of a *KRAS* mutation (*KRAS* ^mut^), PCR products were extracted using the MinElute Gel extraction kit (QIAGEN), and cloned into the pGEM-T Easy vector system (Promega, Fitchburg, WI). Five clones were subsequently selected from each CTC specimen and 5 from each tumor specimen, and all 10 clones were sequenced (GATC Biotech®, Konstanz, Germany).

Droplet digital PCR (ddPCR) was used to validate selected mutations in *KRAS* p.G12D, *KRAS* p.G12V, *KRAS* p.G12S, *KRAS* p.G12C, *KRAS* p.G13D, and *KRAS* p.G13S. Four tumor-CTC “DNA pairs”, 3 tumor DNA specimens and 7 CTC DNA specimens were analyzed with confirmatory ddPCR. Each assay was performed four times using a Bio-Rad QX100 ddPCR system as described previously^32^. Mutations-specific ddPCR assays for detection of the *KRAS* mutations mentioned above were designed in-house. For the *KRAS* mutations, a dual-labeled locked nucleic acid (LNA) probe strategy was used with FAM and HEX as fluorescent dyes. For details of the analysis please see the supplementary material. Briefly, the raw fluorescence amplitude was analyzed using the Quantasoft version 1.6.6 software and used to obtain the fractional abundance for a given mutation. This was reported as the allele frequency. For calculation of the allele fraction the total number of droplets (with and without DNA) was used to calculate DNA copies/ml. For this, we divided the number of mutant copies by the number of total DNA copies (mutant plus wild-type), and multiplied by 100 to give the percentage (allele fraction) of mutant DNA copies.

### Statistical analysis

Statistical evaluation was performed using Graphpad Prism 6 for MAC OS X (GraphPad Software, La Jolla California USA) with p values of p < 0.05 considered significant. The association of CTCs and the different subgroups in correlation with individual clinical characteristics, including T-stage, nodal involvement, resection status, UICC classification and operation technique, were compared using one-way ANOVA, Fisher´s exact test or chi-square test. Univariate survival analysis for CTC positivity, *KRAS* mutation status, and different subgroups of CTC features were performed using the Kaplan-Meier method and compared using the log-rank (Mantel-Cox) test. Survival was calculated from the time of study enrollment to death of the patient. Patients who were still alive at the end of the study period (February 2016) were censored. Within each ddPCR experiment, we ran 4 wells of wild-type (WT) DNA only control (human genomic DNA digested with HaeIII). A 95% confidence interval of false-positive mutant droplets was calculated for each mutation after pooling control wells from individual plates. Using 2-tailed unpaired t-test, we compared mutant droplet counts of a given sample to assay-specific false positives. This to our best knowledge reflects common practice of ddPCR interpretation (Rare Mutation Detection Best Practices Guidelines [Internet]. Feb 2015, Available: http://www.bio-rad.com/webroot/web/pdf/lsr/literature/Bulletin_6628.pdf, accessed 24 April 2017).

## Results

### Patient characteristics

We analyzed the blood specimens of 58 patients with histologically-proven PDAC (median age 67 years, range 41–92 years) for CTCs between February 2012 and June 2014.

Tumor stages varied from small node negative UICC IA tumors to metastatic stage IV disease (Table 1). Thirty-seven of the 58 (63.8%) patients underwent pancreatic resection, and 21 (36.2%) were bypassed. Forty-seven patients received adjuvant or palliative chemotherapy. The median follow-up time was 24 months for living and 10 months for deceased patients. Thirteen patients were alive at the end of the study period. Tissue samples were available from 37 patients. Three of the 10 control-blood samples were from healthy donors (Table 1).

**Table 1:** Demographics, tumor characteristics, circulating epithelial cell status, and median survival of patients with PDAC (n = 58) and control patients (n = 10).

Parameter	All	CTC *KRAS* ^G12V^	CTC *KRAS* ^*other*^ mutation	Cyto positive *KRAS* ^*WT*^	CTC Negative	*P*
N	68	14	28	11	15/5	
PDAC patients, n (%)	58	14 (24%)	28 (48%)	11 (19%)	5 (9%)	**<0**.**0001**
Control group, n (%)	10	0 (0%)	0 (0%)	0 (0%)	10 (100%)	
Median age, years	67	68	67	69	65	0.88
T-Stage						
T1, n (%)	2 (3.5%)	0 (0%)	1 (1.7%)	0 (0%)	0 (0%)	0.409
T2, n (%)	3 (5.2%)	1 (1.7%)	2 (3.4%)	0 (0%)	0 (0%)	
T3, n (%)	30 (51.8%)	6 (10.4%)	15 (25.9%)	5 (8.6%)	4 (7%)	
T4, n (%)	1 (3.4%)	0 (0%)	1 (1.7%)	0 (0%)	1 (1.7%)	
Tx, n (%)	19 (37.9%)	7 (12%)	9 (15.5%)	6 (10.4%)	0 (0%)	
N-Stage						
N0, n (%)	8 (13.8%)	3 (5.2%)	5 (6.9%)	0 (1.7%)	0 (0%)	0.376
N1, n (%)	28 (48.3%)	4 (6.9%)	14 (24.1%)	5 (8.6%)	5 (8.6%)	
Nx, n (%)	22 (37.9%)	7 (12%)	9 (15.5%)	6 (10.4%)	0 (0%)	
Resection margin						
R0, n (%)	28 (48.3%)	6 (10.4%)	14 (24.1%)	5 (8.6%)	3 (5.2%)	0.275
R1, n (%)	8	1 (1.7%)	4 (6.9%)	1 (1.7%)	2 (3.4%)	
Not resected, n (%)	22 (37.9%)	7 (12%)	9 (15.5%)	6 (10.4%)	0 (0%)	
UICC classification						
IA, n (%)	1	0 (0%)	1 (1.7%)	0 (0%)	0 (0%)	0.549
IB, n (%)	2	1 (1.7%)	0 (0%)	1 (1.7%)	0 (0%)	
IIA, n (%)	4	1 (1.7%)	3 (5.2%)	0 (0%)	0 (0%)	
IIB, n (%)	28	5 (8.6%)	14 (24.1%)	5 (8.6%)	4 (6.9%)	
III, n (%)	11	4 (6.9%)	3 (5.2%)	1 (1.7%)	1 (1.7%)	
IV, n (%)	12	3 (5.2%)	6 (10.4%)	5 (8.6%)	0 (0%)	
Operation technique						
PPPD/Whipple, n (%)	28 (48.3%)	6 (10.3%)	13 (22.4%)	6 (10.4%)	3 (5.2%)	0.149
Bypass/ Exploration, n (%)	21 (36.2%)	6 (10.4%)	9 (15.5%)	5 (8.6%)	1 (1.7%)	
Left resection, n (%)	9 (15.5%)	2 (3.5%)	5 (8.6%)	1 (1.7%)	1 (1.7%)	

*UICC* Union international contre le cancer, *n*.*s*. not significant.

### Genetic and cytologic detection of PDAC cell lines spiked in whole blood

Whole blood to which PDAC cells were added (spiked) was used to determine the sensitivity of CTC detection by cytology and *KRAS* mutational capture analysis. PANC-1 cells that are known to contain a *KRAS* ^G12D^ mutation were spiked into whole blood to final concentrations of 10, 2 and 1 cells/ml. Cytological evaluation revealed the PANC-1 cells in all samples (n = 6) spiked with 10 cell/ml and was able to detect PANC-1 cells in 1 of 2 specimens spiked with 2 cells/ml or 1cell/ml respectively (Supplementary Table 1). Using mutant *KRAS* as a molecular biomarker for the presence of CTC, *KRAS* mutations were detected in all spiked samples (n = 16), including at concentrations as low as 1 cell/ml (Supplementary Table 1).

### CTC counts and survival

Thirty-nine patients (67.3%) showed CTC clusters or single CTCs based on cytomorphology (Fig. 1a,b), 2 (3.4%) had cytology suspicious for CTCs and 17 (29.3%) were cytologically negative for CTC. Inter-observer consensus was high with a κ- value of 0.82 (PB vs. ST), 0.92 (PB vs. MBP) and (ST vs. MPB) 0.89 respectively (p > 0.001). Initial disagreement was present in 5 cases, and a majority decision was performed.

The total number of visible, malignant appearing CTCs on cytology ranged from 0–13 CTC/ml. In 42 of the patients (72.4%), we found ≥1 *KRAS* mutation(s) in the CTC (Tables 1 and 2). Twenty-eight (48.3%) patients had both, a CTC- positive cytologic specimen and ≥1 *KRAS* mutation(s) in the CTC. Five (8.7%) specimens showed neither a *KRAS* mutation nor a positive cytologic result. None of the 10 control blood samples had CTC on cytology or a *KRAS* mutation.

**Table 2:** Pairwise comparison of *KRAS* mutations and overall survival in PDAC patients.

Parameter	CTC *KRAS* ^G12V^	CTC *KRAS* ^*other*^ mutation	CTC cyto positive *KRAS* ^*WT*^	CTC Negative
n = 58	14 (24.1%)	28 (48.3%)	11 (19%)	5 (8.6%)
CTC *KRAS* ^G12V^		*P* = 0.21	***P*** = **0**.**04**	*P* = 0.26
CTC *KRAS* ^*other*^ mutation	*P* = 0.21		*P* = 0.23	*P* = 0.90
CTC positive *KRAS* ^*WT*^	***P*** = **0**.**04**	*P* = 0.23		*P* = 0.67
CTC Negative	*P* = 0.26	*P* = 0.90	*P* = 0.67	
Median OS, months	24.5	10	8	8
*P*	0.63			
Alive at end of study period (2/2016)	3	8	2	1

**Figure 2:**
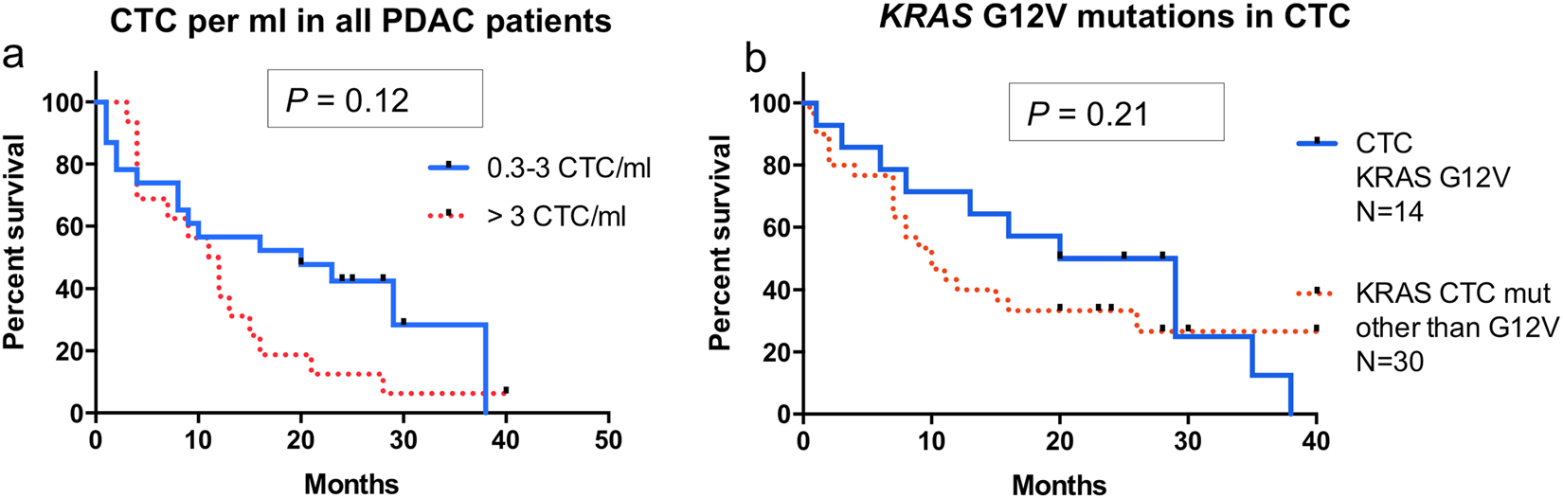
CTC and overall survival. (**a**) Survival analysis of PDAC patients with 0.3–3 CTC in the blood (blue, N = 23, MS 20 months) versus those with >3 CTC/ml blood (red dots, N = 16, MS 11.5 months; *P* = 0.12). (**b**) Survival analysis of PDAC patients with CTC containing a *KRAS* ^G12V^ mutation (MS 24.5 months) and other *KRAS* mutations (MS 10 months; *P* = 0.21).

The mere cytological presence of CTCs had no influence on overall survival (*P* = 0.23; median overall survival, 12 vs 8 months). However, higher numbers of CTC/ml blood were associated with a trend for shorter overall survival. Patients with more than 3 CTC/ml blood (n = 16) had a median overall survival of 11.5 months and patients with 0.3–3 CTC/ml blood (n = 23) of 20 months (*P* = 0.12; Fig. 2).

### Distribution of *KRAS* mutations in CTC and primary tumor

In the *KRAS* mutation analyses of the CTC and primary tumor specimens, we detected various types of codon 12 and 13 mutations (Table 3). The distribution of *KRAS* mutations in the CTC equals that of the mutations in the primary tumor, but CTC also showed rare KRAS mutations (Table 3). Mutations were present in 97.4% (n = 37) of the available 38 tissue samples. The samples mainly harbored c.35G > A (p.G12D; *KRAS* ^G12D^) mutations: they were found in 57.8% of the samples (n = 22). The second most frequent mutation was c.35G > T (p.G12V; *KRAS* ^G12V^) which was found in 16 of the 38 samples (42.1%). Multiple mutations were present in seven tumor specimens and eleven CTC specimens. We only observed overlap of these two groups in one patient (Table 3).

**Table 3:** *KRAS* mutation status of the primary tumor and the CTC DNA in 58 patients; in 20 cases no primary tissue was available.

*KRAS* mutation status	CTC DNA Sample (n = 58)	Primary Tumor Sample (n = 38)
Wild type (GGTGGC) (CTC cyto positive)	11	1
All *KRAS* mutations*	57	44
*KRAS* ^G12V^ (GTT)*	14	16
*KRAS* ^G12D^ (GAT)*	21	22
*KRAS* ^G13S^ (AGC)*	5	2
*KRAS* ^G13D^ (GAC)*	8	4
*KRAS* ^G12C^ (TGT)*	2	—
*KRAS* ^G12S^ (AGT)*	6	—
*KRAS* ^G12A^ (GCT)*	1	—
multiple *KRAS* mutations	11	7

*Eleven patients had multiple mutations – all mutations are shown here; 6 patients with a *KRAS* ^G12D^ mutation had one additional mutation, and 3 patients with a *KRAS* ^G12V^ mutation had one additional mutation in their CTC. 4 patients had a combination of a *KRAS* ^G12V^ and a *KRAS* ^G12D^ mutation in the primary tumor.

**Figure 3:**
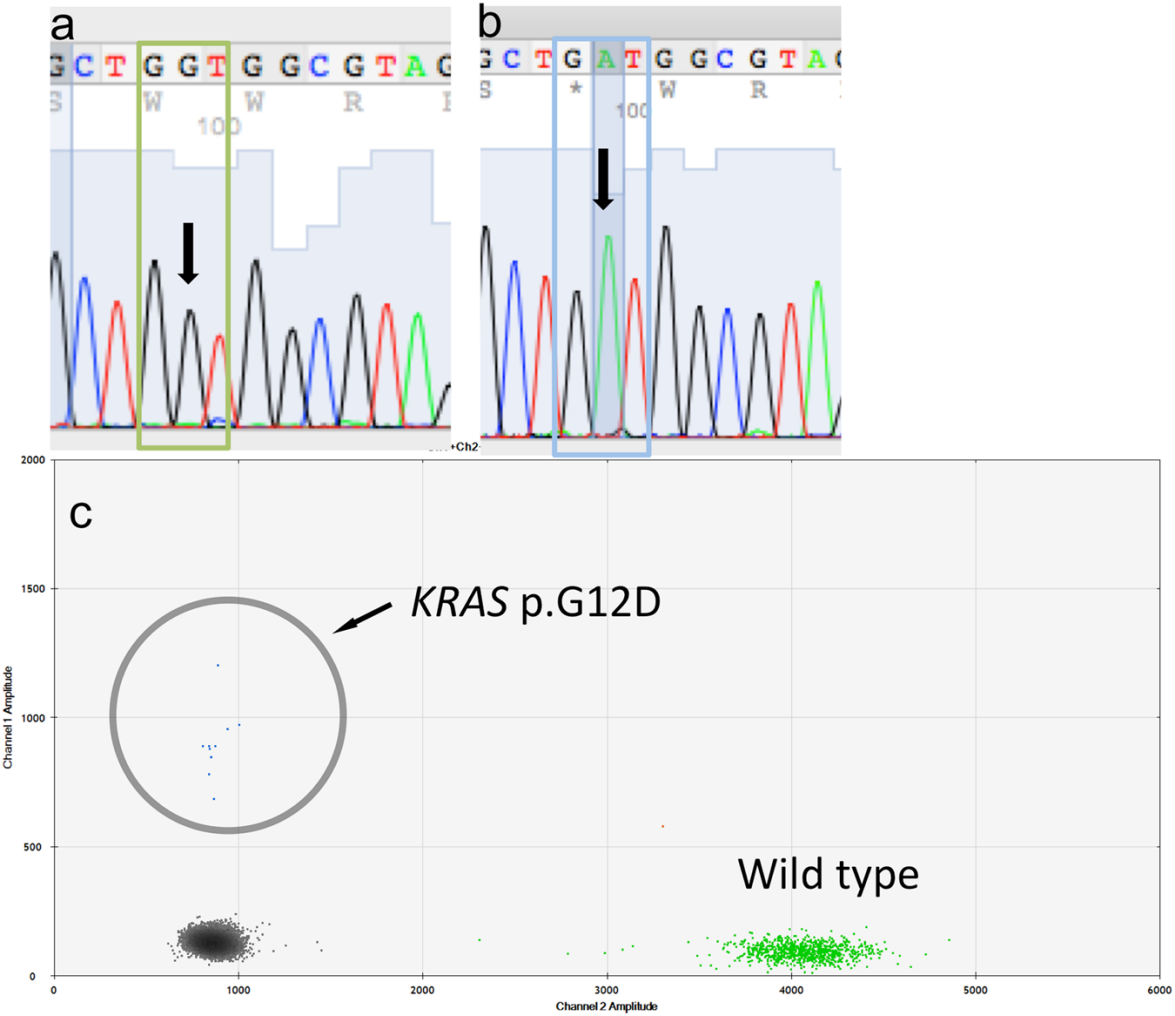
(**a**) Example Chromatogram (antisense strand) wild-type *KRAS* in a negative control sample. (**b**) Example Chromatogram (antisense strand) *KRAS* c.35G > A (pG12D) in a CTC sample. (**c**) Example ddPCR result (Patient 45) for *KRAS* ^G12D.^ The number of events is very low which represents a very low copy of mutant *KRAS* ^G12D^ molecules in the specimen. The circle encircles the area of interest and the approximately 6 events.

**Figure 4:**
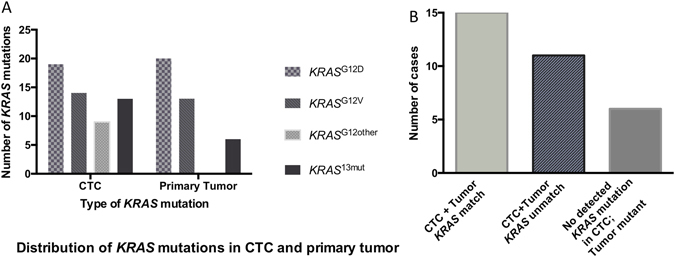
Distribution of *KRAS* mutations in CTC and the primary tumor. (**a**) *KRAS* mutations in CTC and the primary tumor: Several patients (n = 13) had multiple mutations in the CTC or primary tumor, in 20 cases no tissue was available. (**b**) Substantial discordance of *KRAS* mutations in CTC and the primary tumor (n = 9): those with no available tissue samples were excluded (n = 20); 5 were CTC negative. Cyto pos = cytology positive; MS = median survival (in months).

The *KRAS* mutational analysis of CTCs revealed a c.35G > A (p.G12D; *KRAS* ^G12D^) mutation in 21 specimens (Fig. 3), a c.35G > T (p.G12V; *KRAS* ^G12V^) mutation in 14 specimens, and other *KRAS* mutations (c.35G > C (p.G12A), c.34G > T (p.G12C), c.34G > A (p.G12S), c.38G > A (p.G13D), c.37G > A (p.G13S)) in 22 specimens. Eleven patients had more than one *KRAS* mutation in their CTC. Two had a *KRAS* ^G12V^ mutation and were assigned to the *KRAS* ^G12V^ group, five had a *KRAS* ^G12D^ and one additional “other mutation” and were included into the *KRAS* ^other^ group, and four had multiple *KRAS* ^other^ mutations in their CTC (Table 3 and Fig. 4a). All patients with *KRAS* Non-G12V mutations are summarized as *KRAS* ^other^. The frequencies of the varying mutations are in line with those previously described in PDAC (ref.^33^; Table 3). Five patients with available tumor samples harboring *KRAS* mutations did not show CTCs in the blood specimens. Among the patients with *KRAS* ^other^ mutations, those with *KRAS* ^G12D^ had a median overall survival of 9 months (n = 21). Survival analysis additionally revealed 8 months for patients with no detectable *KRAS* mutation in their CTC but positive CTC cytology (*KRAS* ^wt^, n = 11), 10 months for patients with *KRAS* ^other^ mutations including *KRAS* ^G12D^ (n = 30) and 9 months for patients without detected CTC. Patients with *KRAS* ^G12V^ had the longest median overall survival with 24.5 months (n = 14); this was however not statistically significant on overall comparison (*P* = 0.63; Table 2). In pairwise comparison of patients with *KRAS* ^G12V^ and those with *KRAS* ^wt^ (but CTC in the parallel cytology specimen), the overall survival was significantly longer among patients with a *KRAS* ^G12V^ mutation (*P* = 0.04, Fig. 2b, Table 2).

### Substantial discordance in *KRAS* mutations in CTC and primary tumor

Of the 38 patients with *KRAS* information on both the CTCs and the tumor, one was *KRAS* wild-type in the primary tumor, and five patients had no detectable CTC, resulting in 32 tumor-CTC “pairs”: Six patients had no detected *KRAS* mutation in the CTCs (e.g. due to detection limitations), but in the primary tumor (18.7%). Of the 26 patients with detected mutations in both the CTC and the primary tumor, 15 (58%) patients had at least one matching mutation (CTC + Tumor *KRAS* match), while in 11 (42%) patients, the *KRAS* mutations differed between the CTCs and the tumor (CTC + Tumor *KRAS* unmatch; Fig. 4). Eighteen selected tumor and CTC specimens - mainly with multiple mutations - were additionally analyzed with the quantitative droplet digital PCR (ddPCR) method to confirm these heterogeneous results. In the selected cases with multiple mutations all mutations were confirmed in ddPCR, revealing extraordinary low allele-frequencies of <0.03%. It is of note, that the performed t-tests showed not significant results in about half of the cases (Table 4, Fig. 3c, Supplementary Table 2); ddPCR did however not detect all mutations in the analyzed CTC specimens (Table 4, Supplementary Table 2). Interestingly, four additional KRAS mutations were identified by the ddPCR technique (two tumor specimens, two CTC specimens).

**Table 4:** Comparison of KRAS mutation results in clamping PCR and ddPCR in selected patient samples, mainly with multiple mutations in CTC or primary tumor.

Patient Sample ID	Tumor/CTC	KRAS mutation (codon 12/13) in clamping PCR	KRAS mutation (codon 12/13) in ddPCR	Allele fraction (ddPCR): % of mutant DNA
2/6T	Tumor	*KRAS* ^G12D^ (GAT),	***KRAS*** ^**G12D**^ (**GAT**),	*43*.*6*
		*KRAS* ^G13D^ (GAC)	(*KRAS* ^G12V^ (GTT))	*6*.*7*
2/6CTC	CTC	*KRAS* ^G12V^ (GTT)	(*KRAS* ^G12V^ (GTT))	(*0*.*09*)
			(*KRAS* ^G12D^ (GAT))	(*0*.*1*)
2/33T	Tumor	*KRAS* ^G13D^ (GAC)	***KRAS*** ^**G13D**^ (**GAC**)	*0*.*13*
2/33CTC	CTC	*KRAS* ^G12D^ (GAT)	(*KRAS* ^G12D^ (GAT))	*0*.*18*
			(*KRAS* ^G13D^ (GAC))	*0*.*02*
2/40T	Tumor	*KRAS* ^G12D^ (GAT)	***KRAS*** ^**G12D**^ (**GAT**)	*20*.*5*
2/40CTC	CTC	*KRAS* ^G12D^ (GAT)	***KRAS*** ^**G12D**^ (**GAT**)	*2*.*5*
2/45T	Tumor	*KRAS* ^G12D^ (GAT),	***KRAS*** ^**G12D**^ (**GAT**),	*7*.*2*
		*KRAS* ^G12V^ (GTT)	(*KRAS* ^G12V^ (GTT))	*1*.*15*
			(*KRAS* ^G13S^ (AGC)),	*4*.*7*
2/45CTC	CTC	*KRAS* ^G12V^ (GTT),	(*KRAS* ^G12V^ (GTT)),	*0*.*7*
		*KRAS* ^G13S^ (AGC)	(*KRAS* ^G13S^ (AGC)),	*0*.*5*
			(*KRAS* ^G12D^ (GAT))	*1*.*3*
2/12T	Tumor	*KRAS* ^G12D^ (GAT),	***KRAS*** ^**G12D**^ (**GAT**),	*4*.*1*
		*KRAS* ^G12V^ (GTT)	***KRAS*** ^**G12V**^ (**GTT**)	*8*.*7*
2/18T	Tumor	*KRAS* ^G12D^ (GAT),	***KRAS*** ^**G12D**^ (**GAT**),	*23*.*2*
		*KRAS* ^G13D^ (GAC)	***KRAS*** ^**G13D**^ (**GAC**)	*0*.*16*
2/30T	Tumor	*KRAS* ^G12D^ (GAT),	***KRAS*** ^**G12D**^ (**GAT**),	*36*.*2*
		*KRAS* ^G12V^ (GTT)	***KRAS*** ^**G12V**^ (**GTT**)	*17*.*7*
2/14CTC	CTC	*KRAS* ^G13S^ (AGC),	*KRAS* ^G13S^ (AGC),	*1*.*1*
		*KRAS* ^G12V^ (GTT)	***KRAS*** ^**G12V**^ (**GTT**),	*2*.*6*
1/26CTC	CTC	*KRAS* ^G13S^ (AGC)	(*KRAS* ^G13S^ (AGC))	*0*.*12*
1/28CTC	CTC	*KRAS* ^G12D^ (GAT),	(*KRAS* ^G12D^ (GAT)),	*0*.*03*
		*KRAS* ^G13D^ (GAC)	(*KRAS* ^G13D^ (GAC))	*0*.*03*
1/32CTC	CTC	*KRAS* ^G12S^ (AGT)	(*KRAS* ^G12S^ (AGT))	*0*.*10*
2/34CTC	CTC	*KRAS* ^G13D^ (GAC),	***KRAS*** ^**G13D**^ (**GAC**),	*3*.*3*
		*KRAS* ^G12S^ (AGT)	(*KRAS* ^G12S^ (AGT))	*0*.*5*
2/50CTC	CTC	*KRAS* ^G12D^ (GAT),	***KRAS*** ^**G12D**^ (**GAT**)	*42*.*7*
		*KRAS* ^G12C^ (TGT)	(*KRAS* ^G12C^ (TGT))	*3*.*9*
2/56CTC	CTC	*KRAS* ^G12C^ (TGT)	(*KRAS* ^G12C^ (TGT))	*0*.*04*
Spiking 2 cells/ml	“CTC”	*KRAS* ^G12D^ (GAT)	***KRAS*** ^**G12D**^ (**GAT**)	*1*.*9*

Statistically significant results are displayed in bold type; statistically non-significant results are displayed in parentheses. ddPCR: digital droplet PCR.

## Discussion

In the present study we were able to isolate and genetically characterize CTC in all stages of PDAC from UICC stage IA to metastatic stage IV patients with a combination of cytological and genetic evaluation. Patients with a higher tumor burden in the blood (>3CTC/ml) showed a trend to poorer OS (11.5 months vs 20 months). The CTC and the primary tumors had equally-distributed genotypes of *KRAS* mutations, but CTC showed more diverse *KRAS* mutations. Surprisingly, the *KRAS* ^G12V^ mutation in CTC was associated with better OS (median OS 24.5 months) compared to *KRAS* ^wt^ (*P* = 0.04). Other mutations, and also CTC negative patients, had similar median overall survival times (8–9.5 months).

It is of note that of the 21 patients with *KRAS* mutations in the CTC and the primary tumor, only 58% had a matching mutation in CTC and tissue. The other 42% had discordant mutations. The “matching” status had no influence on patient overall survival. Additionally, 6 patients with a KRAS mutant tumor and a positive cytology specimen did not show a *KRAS* mutation. This may be due to a detection limit of the *KRAS* assay, or the cytological “positive” CTC were not tumor derived. Moreover, cytological interpretation of CTC specimens can be challenging and depends on the investigator’s experience. Furthermore, benign pancreatic lesions may also shed cells with morphological features similar to those of CTCs into the blood stream^34,35^. All this may lead to false positive or negative results. However, in the present study we had highly concordant scores for the patient samples from three independent and experienced investigators.

Increasing evidence suggests that characterization of CTC from cancer patients may provide important information regarding early detection, prognosis, treatment and relapse as well as improved mechanistic insight with respect to tumor invasion and metastasis. CTC are extensively investigated in breast^5,10^, lung^11^ colorectal^6,12^ and prostate cancer^13,14^. In selected studies in these entities, CTC are used for response prediction and therapy adjustment^5,14^. In PDAC we are far from this scenario, due to inconsistent detection, isolation and characterization of CTC in PDAC^23^. Several studies with the FDA-approved CellSearch® technique were rather disappointing in PDAC patients, with CTC detection rates of about 5^21^–40%^22–24^ but ongoing research has developed more promising approaches^16–19^.

Several studies, including our own, have shown that CTC in PDAC patients are not only rare, but also a diverse population with respect to (EMT) surface markers^17–19^. We were now able to show that *KRAS* mutations are also heterogeneous in this cell population. The CTC showed *KRAS* ^G12V^, *KRAS* ^G12D^, *KRAS* ^G12S^, *KRAS* ^G13D^ and other *KRAS* mutations. Additionally, not only the CTC but also the primary tumors showed samples with more than one *KRAS* mutation. A recent extensive study on distinct tumor subtypes in PDAC revealed similar results: mutations (not only *KRAS*) were partially diverse in the primary tumor and metastatic sites. The authors also describe a high inter-tumor diversity between patients^36^.

*KRAS* mutations in primary tumor samples and metastasis in colorectal cancer have been described as highly similar with a concordance of 93–97%^37,38^. Especially in colorectal cancer, however, where the *KRAS* mutation status is crucial for the decision of anti-EGFR treatment, there is increasing evidence for a paradigm shift. Tumors are described as heterogeneous and may harbor small subsets of cells with specific mutations not found in routine diagnostics, showing heterogeneous mutations and may thus limit - or direct eligibility for anti-EGFR treatment^39–41^. Additionally, *KRAS* mutations can be reliably found in CTC of colorectal patients with a robust concordance of mutations of 44–77% and also 27–56% discordance in small sample sizes (9–12 samples)^39,40^. It is possible that the discordance between primary tumor and CTC could account for the failure of anti-EGFR therapy in patients with *KRAS* ^WT^ tumors, who in fact harbor *KRAS* mutations in their CTC. The only published study on PDAC and *KRAS* mutations in the CTC showed a 100% concordance in the 5 tested sample pairs of CTC and primary tumor^15^.

There are possible biological and technical explanations for the 42% discordant *KRAS* mutations in the CTC and the primary tumor in this study. First, the discordant CTC could represent the cells in transit that may have departed the primary lesions before the acquisition of a fully-malignant phenotype to undergo somatic mutations or deletions at a distant site^20,31,42^. Second, the heterogeneity of *KRAS* mutation status within the primary tumor is a well-known phenomenon^43–45^. In fact, there could thus be more than one *KRAS* mutation in each PDAC of which one “matches” the tumor: Heterogeneous *KRAS* mutations within one tumor were found in 7 of 32 tumor samples. Third, technical limitations like cross-contaminated specimens are theoretically possible- although this is unlikely in 42% of the cases. Additionally, 18 exemplary samples were quantitatively measured by ddPCR and show results very similar to those obtained with the clamping PCR. Finally, discordance may in theory be explained by metastases from a non-detected second primary. It is of note that the used *KRAS* detection method with the PNA Clamp as well as the ddPCR has a detection limit and may lead to false-negative *KRAS* mutational analysis results, as mentioned above. The majorities of mutations that were found in ddPCR had a very low abundance and were partially not statistically significant.

In the present study, we found that patients with the specific *KRAS* ^G12V^ mutation in their CTC had a significantly better OS than patients without detectable *KRAS* mutations in the specimens (*KRAS* ^WT^;(p = 0.04)). In a study on unresectable PDAC, *KRAS* mutations in general were negative predictors for survival, but *KRAS* ^G12V^ mutations were associated with better survival when found in FNA samples^46^. Another study also showed superior survival of the *KRAS* ^G12V^ subtype in resected PDAC samples. The *KRAS* mutation subtypes in that study had a higher impact on survival than molecular factors such as p53, p16^INK4^, p21^WAF1^, and cyclin D1, while the *KRAS* mutations per se were not a risk factor for poor outcome^47^. Other studies in colorectal cancer showed inferior survival in patients with *KRAS* ^G12V^ ^48^.

The underlying biological phenomenon remains unclear to date; the success of different chemotherapies, however, may also depend on the identification of a unique genotypic pattern. The *KRAS* mutation phenotype (mutant or wild-type) has been used successfully for treatment decisions for anti-EGFR treatment in colorectal cancer: patients with *KRAS* mutations benefited significantly less from anti-EGFR treatment than patients with *KRAS* wild-type tumors^49^. Recently, however, the specific genotype of *KRAS* ^G13D^ showed better response to treatment than other *KRAS* mutations^50,51^. Tumor heterogeneity may confound chemotherapeutic strategies in personalizing care for individual patients, and a more complete picture of the cancer cells inside an individual may help treatment decisions. To date, mutant *KRAS* is considered an undruggable target^52^, there is however ongoing research in this field. New approaches for blocking *KRAS* activity continue to be developed and further insights into the biology of the specific *KRAS* mutations of primary tumors and CTC may provide better therapeutic targets in the future^53,54^.

The present study has several limitations. The CTC isolation-by-size is independent of surface markers but may miss rather small but potentially relevant CTC; additionally, the CTC specimens have to be read by a trained cytologist to address the nuances of cell cytomorphology. The method has, however, been found to isolate more CTC than surface antigen-dependent methods such as CellSearch®^19^. It is of note that the used *KRAS* detection method with the PNA clamp as well as the ddPCR has a detection limit and may lead to false-negative *KRAS* mutational analysis results. High numbers of CTCs isolated on the parallel cyto-filter however suggests that this is not the case in these patients (data not shown). The used *KRAS* PCR with the PNA Clamp is an allele-specific blocker (ASB) PCR. Its superiority has, however, been described especially in CTC-mutation detection compared to methods that use more advanced techniques like low denaturation temperature PCR (TransgenomicTM)^55^. One may argue that newer methods, like digital droplet PCR (ddPCR), would lead to better results; this was however not the case in 18 exemplary specimens, where the clamping PCR found more mutations than the very sensitive ddPCR (Table 4). And although the PNA-PCR is rather “old-fashioned”, we experienced it as a very reliable and sensitive detection method. In previous studies we - and others - found a *KRAS* mutation when we diluted down to two tumor cells per ml blood in spiking experiments^16,56^, and we have now found PANC-1 cells even at final concentrations of 1cell/ml. The consecutive mini-prep additionally allows the detection of multiple mutations after direct sequencing. Furthermore, the proposed study includes only 58 patients and the results - especially with respect to survival analysis and the small group of CTC-negative patients (n = 5) in this study- should be interpreted with caution. Additionally, due to the low number of mutations, even thorough statistical analysis cannot fully exclude artefacts. On the other hand, genuine heterogeneous, partially discordant mutations in CTC and primary tumor samples should not be downscaled by rigorous statistics.

Nevertheless, this is one of the largest studies on CTC in PDAC. And despite its aforementioned limitations this is, to the best of our knowledge, the first study comparing *KRAS* mutation status in 26 matched primary tumors and CTC showing a substantial match but also discordant mutations.

These preliminary results in 58 PDAC patients suggest that a) higher numbers of CTCs per ml may be associated with an inferior outcome, b) the subtype of CTC with a *KRAS* ^G12V^ mutation may be associated with better survival, and c) *KRAS* mutations in the CTC (and the tumor) can be heterogeneous and can be different from the mutations in the primary. The superiority in survival of patients with the *KRAS* ^G12V^ mutation in CTC needs to be evaluated in larger studies. Yet, it appears that a specific mutation may be important in the biology of a cancer, but its effect on chemotherapy and resistance remains to be determined in pancreatic CTC. Finally, the divergence between the disseminated tumor cells and the primary tumor as well as intra-tumoral heterogeneity may be one hypothesis for the poor response to treatment in PDAC and should be investigated in further detail.

## Electronic supplementary material

Supplementary DOC File
