# Author Correction: Pancreatic cancer: Circulating Tumor Cells and Primary Tumors show Heterogeneous *KRAS* Mutations

**DOI:** 10.1038/s41598-017-14870-3

**Published:** 2017-10-27

**Authors:** Birte Kulemann, Stephanie Rösch, Sindy Seifert, Sylvia Timme, Peter Bronsert, Gabriel Seifert, Verena Martini, Jasmina Kuvendjiska, Torben Glatz, Saskia Hussung, Ralph Fritsch, Heiko Becker, Martha B. Pitman, Jens Hoeppner

**Affiliations:** 1 Center for Surgery, Department of General and Visceral Surgery, Medical Center University of Freiburg, Freiburg, Germany; 2 Institute for Surgical Pathology, Medical Center University of Freiburg, Freiburg, Germany; 3 Comprehensive Cancer Center, Medical Center University of Freiburg, Freiburg, Germany; 4 Department of Pathology & Andrew L. Warshaw, MD Institute for Pancreatic Cancer Research, Massachusetts General Hospital/ Harvard Medical School, Boston, MA USA; 5 Center of Medicine, Department of Medicine I, Medical Center University of Freiburg, Freiburg, Germany; 6 Faculty of Medicine, University of Freiburg, Freiburg, Germany

*Scientific Reports* **7**:4510; doi:10.1038/s41598-017-04601-z; Article published online 03 July 2017

In the original version of this Article, size-based CTC-isolation is erroneously referred to as ‘ISET’. ‘ISET’ is a Trademark of Rarecells. The two incidences of ‘ISET’ (in the abstract and the ‘CTC isolation method’ section) have been removed.

