# Supplementary material for: Pancreatic cancer: Circulating Tumor Cells and Primary Tumors show Heterogeneous KRAS Mutations
Source: Sci Rep. 2017 Jul 3;7:4510. doi: 10.1038/s41598-017-04601-z (PMC5495768; doi:10.1038/s41598-017-04601-z)
Supplement: Supplementary file 1 — Supplementary DOC File [file 41598_2017_4601_MOESM1_ESM.doc]

**Supplementary material**

**Pancreatic cancer: Circulating Tumor Cells and Primary Tumors show Heterogeneous KRAS Mutations.**

Birte Kulemann^1,6^, Stephanie Rösch^1,6^, Sindy Seifert^1,6^, Sylvia Timme^2,6^, Peter Bronsert^2,3,6^, Gabriel Seifert^1,6^, Verena Martini^1,6^, Jasmina Kuvendjiska^1,6^, Torben Glatz^1,6^, Saskia Hussung^5,6^ , Ralph Fritsch^5,6^, Heiko Becker^5,6^, Martha B. Pitman^~~4~~^, & Jens Hoeppner^1,3,6^

^1^ Center for Surgery, Department of General and Visceral Surgery, Medical Center University of Freiburg, Germany

^2^Institute for Surgical Pathology, Medical Center University of Freiburg, Germany

^3^Comprehensive Cancer Center, Medical Center University of Freiburg, Germany

^4^Department of Pathology & Andrew L. Warshaw, MD Institute for

Pancreatic Cancer Research, Massachusetts General Hospital/ Harvard

Medical School, Boston, MA, USA

^5^Center of Medicine, Department of Medicine I, Medical Center University of Freiburg, Germany

^6^Faculty of Medicine, University of Freiburg, Germany

**Supplementary table 1.** Molecular Analysis and Cytology Results of Spiking Experiments

|  | 10 cells/ml  (n=6) | 2 cells/ml  (n=6) | 1 cell/ml  (n=4) | negative control  (n=10) | PANC-1 control  (n=10) |
| --- | --- | --- | --- | --- | --- |
| *KRAS* mutation | 100% | 100% | 100% | 0% | 100% |
| Malignant cytology result | 100% | 50% | 50% | 0% | 100% |

Spiking experiments: Specified numbers of PANC-1 cells were added to 3 mL (cytology device) or 6 ml (molecular biology device) of normal blood: 10 cells/ml (n = 6), 2 cells/ml (n = 6), 1cell/ml (n = 4), of healthy blood were tested for cytology results and *KRAS* mutations. Buffy coat and PANC-1 DNA was used as negative and positive controls, respectively.

**Supplementary information for droplet digital PCR (ddPCR)**

Droplet digital PCR (ddPCR) was used to validate selected mutations in *KRAS* p.G12D, *KRAS* p.G12V, *KRAS* p.G12S, *KRAS* p.G12C, *KRAS* p.G13D, and *KRAS* p.G13S. Four tumor-CTC “DNA pairs”, 2 tumor DNA specimens and 6 CTC DNA specimens were analyzed with confirmatory ddPCR. Each assay was performed four times using a Bio-Rad QX100 ddPCR system as described previously ^32^. Mutations-specific ddPCR assays for detection of the KRAS mutations mentioned above were designed in-house. For the *KRAS* mutations, a dual-labeled locked nucleic acid (LNA) probe strategy was used with FAM and HEX as fluorescent dyes. Sequences of the LNA probes were as follows:

KRAS G13D WT

5‘-/5HEX/CTG G+TG +GC+G T+AG GCA A/3IABkFQ/-3‘

KRAS G13D MUT

5‘-/56-FAM/CTG G+TG +AC+G T+AG GCA A/3IABkFQ/-3‘

KRAS G12S WT

5‘-/5HEX/TAC G+CC +AC+C A+GC TCC A/3IABkFQ/-3‘

KRAS G12S MUT

5‘-/5HEX/TAC G+CC +AC+T A+GC TCC A/3IABkFQ/-3‘

KRAS WT

5’-/5HEX/CC+A +C+C+A G+CT C/3IABkFQ/-3‘

KRAS G12D MUT

5’-/56-FAM/CGC +C+A+T +CA+G C/3IABkFQ/-3‘

KRAS G12V MUT

5’-/56-FAM/CG+C C+A+A +CAG +CT/3IABkFQ/-3‘

G12V und G12D have the same WT

Thermal cycling conditions for the KRAS assays were:

| Cycling Step | Temperature, °C | Time | Number of Cycles |
| --- | --- | --- | --- |
| Enzyme activation | 95 | 10 min | 1 |
| Denaturation | 95 | 30 sec | 40 cycles |
| Annealing/extension | 55 (KRAS G12D) | 90 sec |
| Enzyme deactivation | 98 | 10 min | 1 |
| Hold (optional) | 12 | Infinite | 1 |

10-minute hold at 95°C, 49 cycles of 95°C for 30 seconds and then 90 seconds 55°C (p.G12D), 59,4°C (p.G12V), 61.1°C (p.G13S), 62°C (p.G12S & p.G13D) or 59.4°C (p.G12C), respectively. This step was followed by 95°C for 30 seconds and cycles were performed 49 times (p.G12D, p.G12S, p.G12C, p.G13D, p.G13S) or 39 times (p.G12V) according to manufacturer´s instructions. Raw fluorescence amplitude was analyzed using the Quantasoft version 1.6.6 software and used to obtain the fractional abundance for a given mutation. This was reported as the allele frequency. For calculation of the allele fraction the total number of droplets (with and without DNA) was used to calculate DNA copies/mL, then the number of mutant copies was divided by the number of total DNA copies (mutant plus wild-type), and multiplying by 100 to give the percentage (allele fraction) of mutant DNA copies on the basis of Poisson distribution of positive to empty droplets. A two tailed independent t-test was performed.

**Supplementary table 2.** Detailed results of the ddPCR of selected cases

| **Sample** | **KRAS**  **Mutation** | **pos. MUT droplets** | **pos. WT droplets** | **% mutated** | **Accepted droplets** | **volume/sample** | **Assay specific 95% CI (droplets/**  **reaction)** | **p value** |
| --- | --- | --- | --- | --- | --- | --- | --- | --- |
| 2/6T | G12D | 1014 | 1311 | 43.61 | 54070 | 12 uL | 0.58 – 1.92 | <0.0001 |
| 2/6T | G13D | 2 | 1347 | 0.15 | 63186 | 12 uL | -0.13 – 1.13 | 1 |
| 2/6T | G12V | 80 | 1121 | 6.66 | 53296 | 12 uL | 0.05 – 0.95 | <0.0001 |
| 2/6CTC | G12V | 1 | 1053 | 0.09 | 72065 | 12 uL | 0.05 – 0.95 | 0.45 |
| 2/6CTC | G12D | 1 | 1013 | 0.10 | 66732 | 12 uL | 0.58 – 1.92 | 0.09 |
| 2/6CTC | G13D | 0 | 1429 | 0.00 | 62975 | 12 uL | -0.13 – 1.13 | - |
| 2/12T | G12D | 300 | 7090 | 4.06 | 48749 | 8 uL | 0.58 – 1.92 | <0.0001 |
| 2/12T | G12V | 628 | 6604 | 8.68 | 71724 | 8 uL | 0.05 – 0.95 | <0.0001 |
| 2/14CTC | G12V | 29 | 1107 | 2.55 | 65558 | 8 uL | 0.05 – 0.95 | <0.0001 |
| 2/14CTC | G13S | 10 | 922 | 1.07 | 59666 | 8 uL | 1.23 – 4.27 | 0.809 |
| 2/18T | G13D | 13 | 8142 | 0.16 | 56890 | 8 uL | -0.13 – 1.13 | 0.001 |
| 2/18T | G12D | 1995 | 6605 | 23.20 | 55776 | 8 uL | 0.58 – 1.92 | <0.0001 |
| 1/26CTC | G13S | 11 | 9382 | 0.12 | 65843 | 8 uL | 1.23 – 4.27 | 1 |
| 1/28CTC | G12D | 6 | 17890 | 0.03 | 63615 | 4 uL | 0.58 – 1.92 | 0.68 |
| 1/28CTC | G13D | 7 | 24650 | 0.03 | 58104 | 4 uL | -0.13 – 1.13 | 0,1 |
| 2/30T | G12D | 216 | 380 | 36.24 | 68741 | 6 uL | 0.58 – 1.92 | <0.0001 |
| 2/30T | G12V | 71 | 330 | 17.71 | 72246 | 6 uL | 0.05 – 0.95 | <0.0001 |
| 1/32CTC | G12S | 2 | 2064 | 0.10 | 59039 | 8 uL | 0.51 – 1.99 | 0.16 |
| 2/33T | G13D | 22 | 16497 | 0.13 | 58785 | 8 uL | -0.13 – 1.13 | 0.0002 |
| 2/33CTC | G13D | 1 | 4099 | 0.02 | 51341 | 8 uL | -0.13 – 1.13 | 0.56 |
| 2/33CTC | G12D | 6 | 3349 | 0.18 | 55246 | 8 uL | 0.58 – 1.92 | 0.77 |
| 2/34CTC | G13D | 18 | 531 | 3.28 | 44336 | 4 uL | -0.13 – 1.13 | 0.0049 |
| 2/34CTC | G12S | 3 | 554 | 0.54 | 70685 | 4 uL | 0.51 – 1.99 | 0.3898 |
| 2/40T | G12D | 471 | 1831 | 20.46 | 73869 | 8 uL | 0.58 – 1.92 | <0.0001 |
| 2/40CTC 2uL/well | G12D | 5 | 735 | 0.68 | 76109 | 8 uL | 0.58 – 1.92 | 1 |
| 2/40CTC 5 uL/well | G12D | 35 | 1379 | 2.48 | 76309 | 20 uL | 0.58 – 1.92 | 0.049 |
| 2/45T | G12V | 3 | 259 | 1.15 | 63360 | 6 uL | 0.05 – 0.95 | 0.56 |
| 2/45T | G12D | 22 | 285 | 7.17 | 65041 | 6 uL | 0.58 – 1.92 | <0.0001 |
| 2/45T | G13S | 6 | 123 | 4.65 | 73358 | 6 uL | 1.23 – 4.27 | 0.1708 |
| 2/45CTC | G12V | 6 | 844 | 0.71 | 70507 | 8 uL | 0.05 – 0.95 | 0.0795 |
| 2/45CTC | G12D | 11 | 848 | 1.28 | 64639 | 8 uL | 0.58 – 1.92 | 0.1127 |
| 2/45CTC | G13S | 4 | 788 | 0.51 | 65428 | 8 uL | 1.23 – 4.27 | 0.1066 |
| 2/50CTC | G12D | 119 | 158 | 42.96 | 73845 | 4 uL | 0.58 – 1.92 | <0.0001 |
| 2/50CTC | G12C | 1 | 25 | 3.85 | 71836 | 4 uL | 0.00 – 1.25 | 0.3891 |
| 2/56CTC | G12C | 1 | 2707 | 0.04 | 49630 | 8 uL | 0.00 – 1.25 | 0.3891 |
| Spike 2/ml | G12D | 25 | 1329 | 1.85 | 63415 | 12 uL | 0.58 – 1.92 | <0.0001 |
